# Machine Learning-Based Cardiac Output Estimation Using Photoplethysmography in Off-Pump Coronary Artery Bypass Surgery

**DOI:** 10.3390/jcm13237145

**Published:** 2024-11-26

**Authors:** Cecilia A. Callejas Pastor, Chahyun Oh, Boohwi Hong, Yunseo Ku

**Affiliations:** 1 Research Institute for Medical Sciences, Chungnam National University College of Medicine, Daejeon 35015, Republic of Korea; ceciliac@cnu.ac.kr; 2 Department of Otorhinolaryngology-Head and Neck Surgery, Seoul National University Hospital, Seoul 03080, Republic of Korea; 3 Department of Anesthesiology and Pain Medicine, Chungnam National University Hospital, Daejeon 35015, Republic of Korea; 5chahyun@cnuh.co.kr; 4 Department of Biomedical Engineering, Chungnam National University College of Medicine, Daejeon 35015, Republic of Korea; 5 Medical Device Research Center, Department of Biomedical Research Institute, Chungnam National University Hospital, Daejeon 35015, Republic of Korea

**Keywords:** cardiac index, photoplethysmogram, off-pump coronary artery bypass surgery, machine learning, non-invasive hemodynamic monitoring

## Abstract

**Background/Objectives**: Hemodynamic monitoring is crucial for managing critically ill patients and those undergoing major surgeries. Cardiac output (CO) is an essential marker for diagnosing hemodynamic deterioration and guiding interventions. The gold standard thermodilution method for measuring CO is invasive, prompting a search for non-invasive alternatives. This pilot study aimed to develop a non-invasive algorithm for classifying the cardiac index (CI) into low and non-low categories using finger photoplethysmography (PPG) and a machine learning model. **Methods**: PPG and continuous thermodilution CO data were collected from patients undergoing off-pump coronary artery bypass graft surgery. The dataset underwent preprocessing, and features were extracted and selected using the Relief algorithm. A CatBoost machine learning model was trained and evaluated using a validation and testing phase approach. **Results**: The developed model achieved an accuracy of 89.42% in the validation phase and 87.57% in the testing phase. Performance was balanced across low and non-low CO categories, demonstrating robust classification capabilities. **Conclusions**: This study demonstrates the potential of machine learning and non-invasive PPG for accurate CO classification. The proposed method could enhance patient safety and comfort in critical care and surgical settings by providing a non-invasive alternative to traditional invasive CO monitoring techniques. Further research is needed to validate these findings in larger, diverse patient populations and clinical scenarios.

## 1. Introduction

Hemodynamic monitoring is crucial in managing critically ill patients and those undergoing major surgery. It enables timely, targeted interventions by accurately assessing patients’ hemodynamic status, significantly improving clinical outcomes [1,2,3]. While traditional measures like heart rate (HR) and blood pressure (BP) are commonly used, they fall short in fully evaluating organ perfusion, oxygen transport, and identifying the root causes of hemodynamic instability [4]. This limitation has driven the continuous search for more advanced hemodynamic indices, including cardiac output (CO), to provide a more comprehensive understanding of the patient’s condition [5,6].

Despite advances in monitoring technologies, the thermodilution method using a pulmonary artery catheter (PAC) remains the gold standard for CO measurement. However, its invasive nature limits its widespread clinical use, driving demand for non-invasive or minimally invasive alternatives [7,8,9]. Research highlights CO as a vital marker for diagnosing the cause of hemodynamic deterioration and guiding treatment strategies [4,10]. Depending on CO levels, diverse intervention strategies can be employed. In instances of low-output states, such as hemorrhagic shock, the principal aim of hemodynamic intervention is to augment CO. Conversely, in high-output states like early sepsis, management emphasis shifts towards addressing additional factors such as systemic vascular resistance [11,12].

In recent years, the photoplethysmogram (PPG) has evolved beyond its initial use in measuring oxygen saturation and heart rate to become a versatile tool for assessing various biological parameters [13,14,15]. While much research has explored PPG’s potential for blood pressure estimation with promising results [15,16,17], fewer studies have investigated its use in CO estimation [18,19]. Moreover, there is a lack of research using PPG for CO estimation based on a standard reference method (i.e., thermodilution technique) in surgical patients.

This pilot study aims to address this gap by developing a non-invasive algorithm to classify the cardiac index (CI, calculated as CO divided by body surface area) into low-output and non-low-output categories. The algorithm uses finger PPG as the input and continuous CO data from pulmonary artery catheter thermodilution as the reference standard. We use continuous intraoperative recordings from patients undergoing off-pump coronary artery bypass graft (OPCAB) surgery, a group chosen to minimize potential confounding factors common in cardiac surgery, such as those from mechanical circulatory support or hypothermia, which could affect signal quality and interpretation [20]. We employ a machine learning model to develop this classification algorithm, potentially offering a novel, non-invasive approach to CO monitoring in critical care and surgical settings.

By exploring the use of PPG technology and machine learning for cardiac output classification, this study aims to contribute to the ongoing improvement in hemodynamic monitoring techniques. Successfully developing such an algorithm could significantly impact patient care, potentially enhancing safety and comfort by reducing reliance on invasive monitoring methods while maintaining accurate hemodynamic assessment.

## 2. Materials and Methods

### 2.1. Study Design and Participant Selection

This retrospective study included all available intraoperative recordings of patients who underwent OPCAB surgery with continuous thermodilution CO and pulse oximetry (SpO_2_) monitoring under general anesthesia between February 2021 and December 2022. Subjects were excluded based on the following criteria: (1) preoperative echocardiography revealing moderate to severe tricuspid valve regurgitation; (2) preoperative echocardiography showing intra-cardiac shunt lesions; (3) intraoperative central venous pressure waveform or postoperative chest radiography indicating a malpositioned pulmonary artery catheter; and (4) use of mechanical circulatory support during the procedure.

**Figure 1:**
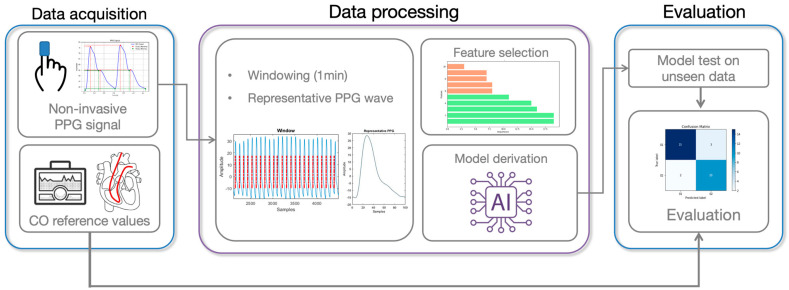
Overview of the proposed methodology for estimating CO from PPG data, including data acquisition, data processing, and evaluation. Abbreviations: CO (cardiac output), PPG (photoplethysmography).

The study protocol received approval from the Institutional Review Board of Chungnam National University Hospital (CNUH 2021-04-090). Informed consent was waived due to the retrospective nature of the study. The overall development process encompassed data acquisition, processing, feature extraction and selection, model derivation, and performance evaluation (Figure 1).

All vital data were obtained from the prospective registry of vital signs for surgical patients at Chungnam National University Hospital (CNUH IRB 2019-08-039). The registry utilizes Vital Recorder (version 1.10, available at https://vitaldb.net (accessed on 23 September 2024), Seoul, Republic of Korea), a freely available data acquisition software designed for recording and analyzing biosignal waveforms and vital signs from various commercial devices [21].

### 2.2. Data Acquisition

Continuous thermodilution CO data were obtained using a Swan-Ganz (7.5 F Swan-Ganz Continuous Cardiac Output Thermodilution Catheter: CCOmbo V, Model 774F75, Edwards Lifesciences, Irvine, CA, USA) and a HemoSphere advanced monitoring platform (Edwards Lifesciences, Irvine, CA, USA). The system estimated and updated CO every 60 s in STAT mode, without employing a moving average process, and recorded at 0.5 Hz. To account for demographic variations, the CI was utilized for model derivation.

Finger PPG signals, acquired from a disposable oximeter sensor (NellcorTM Neonatal-Adult SpO_2_ Sensor, Covidien, Mansfield, MA, USA) attached to either the index or third finger of the subject and a patient monitor (Intellivue MX800, Philips, Boeblingen, Germany), were recorded at a frequency of 125 Hz.

Additional patient data acquired from the electronic medical record included patient age, sex, weight, height, and comorbid conditions. Details on intraoperative utilization of vasoactive/inotropic agents, procedure type (conventional or minimally invasive), and distinct phases of the procedure (harvesting and graft implanting) were also included. The delineation of procedural phases was determined by the timing of systemic heparinization, serving as an approximate indicator of the end of the harvesting phase.

### 2.3. Data Processing

In the preprocessing phase, we conducted a visual inspection to identify and exclude any instances of missing or erroneous data. Both CO and PPG signals were then uniformly re-sampled at 125 Hz. The PPG data were segmented into 1-minute intervals, with each segment overlapping the subsequent one by 10 s. This overlapping strategy, resulting in 7399 windows, was chosen to minimize windowing artifacts and optimize the feature extraction process.

**Figure 2:**
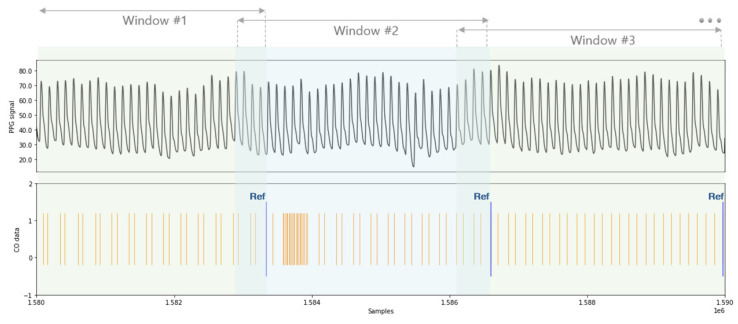
Demonstration of the window division and CO reference value selection process. The PPG signal is divided into windows, and the last CO value of each window is selected as the reference. Abbreviations: CO (cardiac output), PPG (photoplethysmography).

Within each segmented window, a single CO value was selected as the reference. The last recorded CO value within the window’s timeframe was designated as the representative value for that specific window, as depicted in Figure 2. These CO values were then categorized into two groups: values ≤ 2 L/min/m^2^ were classified as “Low CO”, while values > 2 L/min/m^2^ were classified as “Non-low CO”. These CO values were then categorized into two groups: values ≤ 2 L/min/m^2^ were classified as “Low CO”, while values > 2 L/min/m^2^ were classified as “Non-low CO”. This threshold was selected based on an extensive literature review. A CI below 2 L/min/m^2^ is widely recognized as indicating low cardiac output syndrome in cardiac surgery patients, associated with increased mortality and morbidity [22,23]. This value represents a clinically meaningful cutoff where intervention is typically required to prevent organ dysfunction and adverse outcomes.

Following this classification, we applied additional processing to the PPG signal. A second-degree bandpass filter (0.1–15 Hz) was employed, followed by a detrending process to remove the direct current (DC) offset [24,25].

**Figure 3:**
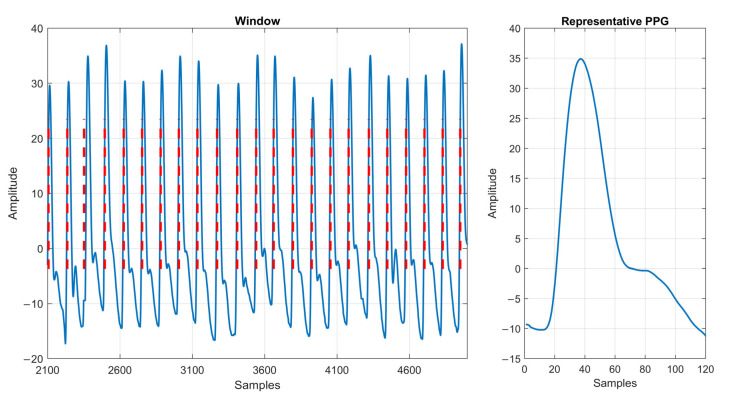
(**Left**) A single PPG window with red vertical lines indicating the detection of each wave. (**Right**) A representative PPG waveform of the window. Abbreviations: PPG (photoplethysmography).

Within each analysis window, multiple PPG waves corresponded to a single CO value. To create a representative PPG waveform for each window, we identified the peaks of all cardiac cycles within the window. These beats were then averaged to generate a composite waveform that characterized the window’s cardiac activity [26]. Figure 3 illustrates this methodology and the resulting representative waveform.

### 2.4. Feature Extraction and Selection

**Figure 4:**
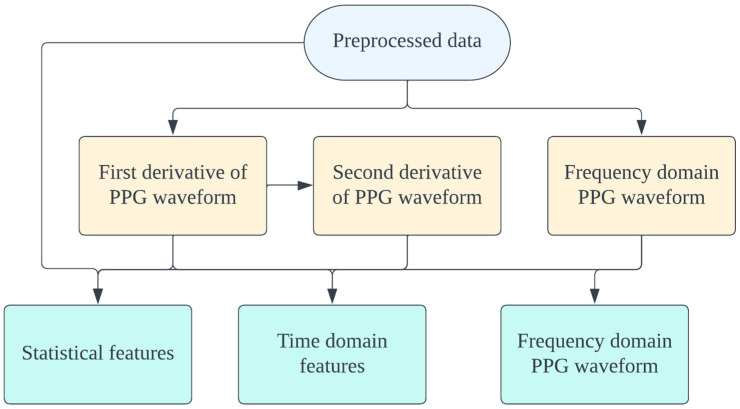
Feature extraction process performed on the PPG signal. Abbreviations: PPG (photoplethysmography).

Figure 4 summarizes the feature extraction process adopted in this study. Each representative PPG waveform contains relevant information, including characteristics of systole, diastole, notch, and pulse width [25,27,28,29]. In addition to the original waveform, we incorporated its first and second derivatives in the feature extraction process.

From these domains, we extracted and selected a total of 20 features using the Relief algorithm, a well-established feature selection method [30,31]. This algorithm identifies conditional dependencies between features and provides a unified view of their importance in an estimated regression model, employing a statistical approach that avoids heuristic search [32].

The Relief algorithm determines the weights of predictors for a continuous target variable by rewarding predictors that give different values to neighbors with different response values and penalizing predictors that give different values to neighbors with the same response value. The algorithm considers two neighbors and assigns weights based on three criteria: (1) the weight of two different values for the response, (2) the weight of two different values for the predictor, and (3) the weight of two different responses for the predictor.

Initially, all weights are set to 0. The algorithm then randomly and iteratively selects an observation and finds its k-nearest neighbors. For each value, the weights are updated. After each iteration, the intermediate value weight is calculated using the following equation:

(1) $${W_{dy}}^{i}={W_{dy}}^{i-1}+{∆}_{y}\left(x_{r,}x_{q}\right)\times d_{qr}\;{W_{dj}}^{i}={W_{dj}}^{i-1}+{∆}_{j}\left(x_{r,}x_{q}\right)\times d_{qr}\;{W_{dy\&dj}}^{i}={W_{dy\&dj}}^{i-1}+{∆}_{y}\left(x_{r,}x_{q}\right)\times {∆}_{j}\left(x_{r,}x_{q}\right)\times d_{qr}$$

After iterating all values, the predictor weight is calculated with the following formula:

(2) $$W_{j}=\frac{W_{dy\&dj}}{W_{dy}}-\frac{{W_{dj}-W}_{dy\&dj}}{m-W_{dy}}$$

where *m* is the total number of iterations performed by the algorithm.

### 2.5. Model Derivation

We divided our dataset into three distinct sets: a training set of 4735 windows (64.0%), a validation set of 1184 windows (16.0%), and an unseen test set of 1480 windows (20.0%). During the training phase, we implemented a robust 5-fold cross-validation method [33].

The specific analytical model employed in this study was CatBoost. The CatBoost model, an abbreviation for Categorical Boosting, is an advanced machine learning algorithm based on gradient boosting over decision trees. It is particularly effective for datasets with numerous categorical features and is known for its ability to handle such features without extensive preprocessing. CatBoost operates by constructing a series of decision trees sequentially, where each subsequent tree corrects the errors of the previous ones.

For classification, the CatBoost model can be represented as follows:

(3) $$\hat{Y}=argmax(\sum_{k=1}^{K}f_{k}(X))$$

Here, $\hat{Y}$ denotes the predicted class, X represents the input features, and $f_{k}$ signifies the k-th decision tree in the ensemble. The ‘argmax’ function selects the class with the highest cumulative vote from all trees. During training, CatBoost optimizes a specific loss function appropriate for classification tasks, typically involving a form of gradient descent, to minimize the classification error [34,35,36].

### 2.6. Statistical Analysis

Continuous variables are expressed as mean ± standard deviation (SD) or median (1Q, 3Q) depending on their distribution. Categorical variables are presented as number (%). Data processing, feature extraction, and model derivation were performed using MATLAB 2022a (Mathworks, Natick, MA) and Python programming language (Python Software Foundation, https://www.python.org/ (accessed on 25 September 2024)). The model’s performance was evaluated using accuracy, which measures the overall correctness of predictions; precision, which quantifies the proportion of true positive predictions; recall (sensitivity), which assesses the ability to identify all positive instances; and F1-score, which provides a balanced measure of precision and recall [37,38,39].

## 3. Results

A total of 75 cases were initially assessed for eligibility. After careful evaluation, eight cases were excluded from the study. The reasons for exclusion were as follows: six cases due to malpositioned PAC, one case due to insufficient recording (absence of SpO_2_ data), and one case identified as an outlier with an excessively high CO range. The excluded outlier case exhibited a CI of 5.0 [4.4, 6.0] L/min/m^2^, which was considered markedly different from the remaining cases and could potentially skew the analysis. The clinical characteristics of the 67 cases included in the final analysis are summarized in Table 1.

### 3.1. Feature Selection Process

The Relief algorithm was employed to select the most informative features from the extracted PPG signal characteristics. This process aimed to identify a subset of features that exhibited the strongest associations with the target variable while minimizing redundancy among the selected features. The algorithm assigned weights to each feature based on their relevance to the target variable, with higher weights indicating greater importance.

Table 2 presents the 20 features selected by the Relief algorithm, along with their descriptions. The selected features encompass a diverse range of PPG signal characteristics, including time-domain, frequency-domain, and non-linear measures. These features provide a comprehensive representation of the PPG signal, enabling the subsequent development of predictive models for estimating CO. By focusing on this subset of informative features, the dimensionality of the input space is reduced, which can improve the efficiency and generalizability of the predictive models [32].

**Figure 5:**
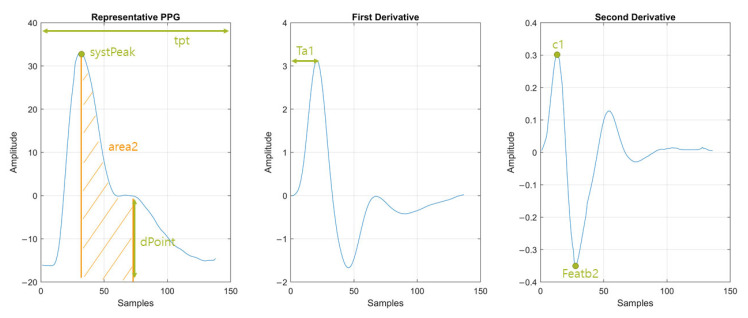
Visual representation of a typical PPG waveform along with its first and second derivatives, highlighting selected features described in Table 2.

Table 2 presents the 20 features selected by the Relief algorithm, along with their descriptions. Figure 5 provides a visual representation of a typical PPG waveform with some of the selected features described in Table 2.

### 3.2. Performance of the Machine Learning Model

The dataset was divided into training and testing sets, with 63.2% of the windows labeled as “Non-low CO” and 36.8% as “Low CO”. This imbalance in the data distribution was taken into consideration during the model’s evaluation and interpretation. The implemented model showed robust performance across all phases. During training and validation, it achieved an average accuracy of 89.42% using 5-fold cross-validation.

**Figure 6:**
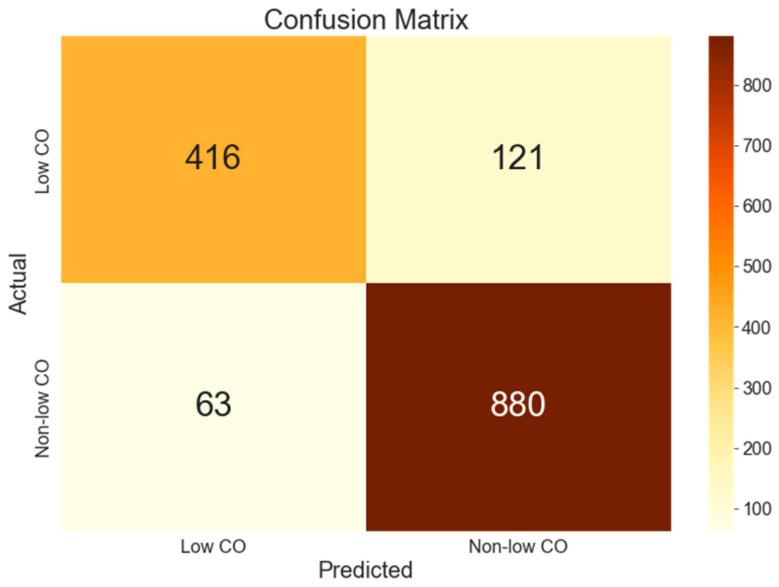
Confusion matrix of the unseen data for “low CO” and “non-low CO” classification.

The model maintained its efficacy in the testing phase, demonstrating an accuracy of 87.57%, as depicted in the confusion matrix shown in Figure 6.

A detailed breakdown of the test set performance by category is presented in Table 3, showing the model’s balanced performance across both CO categories.

Detailed performance metrics revealed balanced capabilities across both CO categories. In the specific context of the “Low CO” category, the model achieved a precision of 0.87 and a recall of 0.77, culminating in an F1-score of 0.82. For the “Non-low CO” category, the precision was 0.88, with a recall of 0.93, resulting in an F1-score of 0.91. These results underscore the model’s proficiency in accurately classifying and differentiating between low and non-low CO states, despite the initial class imbalance.

**Figure 7:**
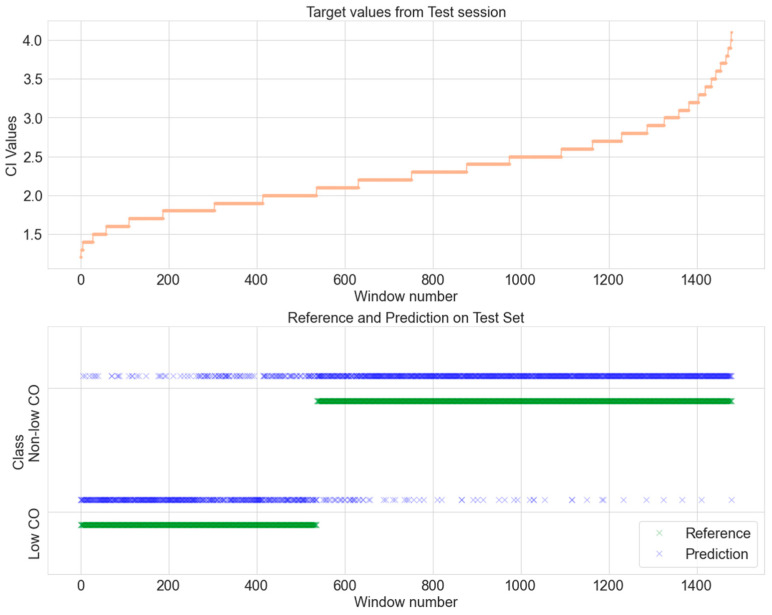
Comparison of actual and predicted values in the unseen data. (**Top**) CI values plotted against the window number. (**Bottom**) Classification of low or non-low CO for both the predictions and references.

In Figure 7, we present a comprehensive comparison between the CO values obtained during the test session and their corresponding reference and predicted values. This figure graphically illustrates the relationship and alignment between the actual CO values and those predicted by the model, providing a clear visual representation of the model’s predictive accuracy.

## 4. Discussion

This pilot study offers valuable insights into the potential of machine learning techniques for non-invasive hemodynamic monitoring, specifically in CO classification using PPG signals. The successful implementation and validation of a CatBoost machine learning model, as evidenced by high accuracy rates in both the validation (89.42%) and testing phases (87.58%), underscores the potential feasibility of using such advanced computational approaches in clinical settings.

A closer look at the model’s performance metrics provides a more nuanced understanding of its capabilities. For the “Low CO” category, the precision score of 0.87 indicates that 87% of instances predicted as “Low CO” were indeed correct. The recall score of 0.77 for this category suggests that the model correctly identified 77% of all actual low CO instances. For the “Non-low CO” category, the precision score of 0.88 and recall score of 0.93 indicate strong performance in identifying normal CO states.

The F1-scores of 0.82 and 0.91 for the “Low CO” and “Non-low CO” categories, respectively, offer a balanced evaluation of the model’s performance. The higher F1-score for the “Non-low CO” category suggests slightly better performance in identifying normal CO states. This difference could be attributed to the data imbalance, with 63.2% of windows labeled as “Non-low CO” and 36.8% as “Low CO”. Despite this imbalance, the model demonstrates robustness in classifying both categories effectively.

Several factors contribute to the CatBoost model’s strong performance. As an ensemble learning method, it combines predictions from multiple decision trees, helping to reduce overfitting and improve generalization [40]. The model employs a novel “ordered boosting” algorithm for constructing decision trees, enabling efficient handling of categorical features and further reducing overfitting [41]. Moreover, its ability to manage complex, non-linear relationships between features and the target variable makes it well suited for CO classification using PPG signals.

Our findings align with previous studies demonstrating the effectiveness of machine learning in non-invasive hemodynamic monitoring. For instance, Kachuee et al. [42] developed a deep learning model for cuffless blood pressure estimation using PPG and ECG signals, achieving promising results. Similarly, Xing et al. [43] employed an SVM classifier for atrial fibrillation detection using PPG signals, showcasing the potential of machine learning in identifying cardiovascular conditions.

As shown in Table 4, our approach achieves competitive performance metrics while using only PPG signals, compared to other methods requiring multiple data sources or more complex measurements. While Xu et al. [44] achieved a good correlation (r = 0.951) using both PPG and arterial pressure waveforms, our method maintains high accuracy (87.57%) with a simpler, single-signal approach. Similarly, Chiu et al. [45] demonstrated a slightly higher accuracy (88.2%) in classifying cardiac output ranges using a Bagged Trees Ensemble model. However, Chiu et al.’s model was trained on a carefully curated subset from the PhysioNet MIMIC-III database, consisting of long-duration, high-quality PPG recordings, which may not be feasible in all clinical settings. Hong et al.‘s study [46], though focused on LCOS prediction rather than continuous CO monitoring, demonstrates the broader applicability of machine learning in cardiac output assessment. Our results suggest that more informative features from preprocessed PPG signals can provide reliable hemodynamic screening when combined with appropriate machine learning techniques, which is comparable to the performance of the previous CO estimation studies.

The model’s high accuracy in the validation phase indicates its robustness, particularly in handling diverse PPG signal features. This can be attributed to the careful preprocessing steps employed in our study, aimed at mitigating the inherent vulnerability of PPG signals to noise. PPG signals are susceptible to various noise sources, including motion artifacts, ambient light interference, and physiological factors like respiration and vasomotor activity [48]. These noise components can significantly affect signal stability and reliability, potentially leading to inaccurate or inconsistent results. To address this challenge, we implemented a comprehensive preprocessing pipeline including signal denoising, outlier removal, and normalization techniques. By meticulously filtering out noise components and ensuring PPG signal integrity, we enhanced the model’s ability to extract meaningful features and make accurate predictions. This approach to PPG signal preprocessing distinguishes our study from previous work and highlights the importance of signal quality control in developing robust machine learning models for non-invasive hemodynamic monitoring.

The model’s commendable accuracy in the testing phase further demonstrates its potential real-world applicability. The balanced F1-scores observed for both “Low CO” and “Non-low CO” categories suggest that the model is equally adept at identifying low and normal cardiac output states, which is crucial for clinical decision making.

While previous studies have demonstrated the utility of PPG signals in monitoring various physiological parameters such as heart rate, blood oxygen saturation, and respiration [42], their application in CO classification has been less explored. Some studies have investigated PPG signals for CO estimation, but with limitations. For instance, Sugo et al. [49] developed a method for estimating CO using PPG signals, but their approach required simultaneous ECG measurements, limiting its practicality in certain clinical settings.

Our study advances the field by demonstrating the feasibility of using PPG signals alone for CO classification, achieving high accuracy without the need for additional sensors. Our PPG-based CO classification method offers several distinct advantages in clinical settings; not only does its non-invasive nature eliminate risks associated with traditional invasive monitoring methods, such as infection, bleeding, or arrhythmias [50,51], but also the single-signal approach reduces the technical complexity and cost compared to multi-signal methods, making it particularly suitable for resource-constrained environments.

However, several limitations should be considered. The method’s accuracy may be affected by factors such as motion artifacts, poor peripheral perfusion, or severe arrhythmias [48]. Additionally, while our binary classification approach provides clinically relevant information, it may not fully capture the granularity of CO changes that continuous numerical measurements provide. Further research with a larger cohort is necessary to validate these findings. Additionally, the study focuses on a specific patient group undergoing OPCAB surgery, and the generalizability of the findings to other patient populations.

From a clinical application perspective, this method shows particular promise in several scenarios. In post-operative monitoring, it could serve as an early warning system for developing low CO states, allowing for timely intervention [52,53]. In intermediate care units, where invasive monitoring might be inappropriate, it could provide valuable hemodynamic information. This method could be especially useful in outpatient cardiac rehabilitation programs or during emergency transport, where continuous, non-invasive monitoring is needed [54].

Looking ahead, several promising directions for future development emerge. Integration with electronic health records could enable automated risk stratification and clinical decision support. Machine learning models could be expanded to predict CO trends, potentially allowing preemptive intervention before clinical deterioration occurs. Furthermore, the methodology could be adapted for specific patient populations or clinical scenarios, such as pediatric patients or pregnancy-related cardiovascular monitoring [55].

Future prospective research should aim to replicate these findings in larger, more diverse populations. Exploring the integration of this machine learning model into real-time monitoring systems could represent a significant advancement, potentially leading to widespread clinical adoption.

The potential implications of this research for clinical practice are substantial. By providing a non-invasive, accurate method for CO classification, it could revolutionize hemodynamic monitoring, particularly in surgical and critical care settings. This would not only enhance patient care but also reduce the risks associated with invasive monitoring techniques.

## 5. Conclusions

This pilot study demonstrates the considerable potential of machine learning techniques and non-invasive PPG signals for accurate CI classification in patients undergoing OPCAB surgery. Our CatBoost model, trained on meticulously preprocessed PPG features, achieved high accuracy rates in both the validation and testing phases, showing balanced performance across low and non-low CO categories.

The successful implementation of this non-invasive approach could markedly enhance patient safety and comfort by mitigating risks associated with invasive monitoring techniques. Our model’s robustness in handling diverse PPG signal features, attributed to our comprehensive preprocessing pipeline, distinguishes this study from previous work. It underscores the critical role of signal quality control in developing reliable machine learning models for non-invasive hemodynamic monitoring.

In conclusion, this study represents a significant advancement in non-invasive hemodynamic monitoring. It highlights the transformative potential of machine learning and PPG signals in clinical practices, particularly in critical care and surgical settings. By offering a non-invasive, accurate method for CO classification, our approach could revolutionize hemodynamic monitoring, ultimately improving patient outcomes and care quality.

## Figures and Tables

**Table 1:** Subject demographic information and characteristics.

Characteristics	Value
Age (yr)	67.0 (59.5, 74.0)
Gender (M)	52 (77.6)
Height (cm)	165.0 (157.1, 169.8)
Weight (kg)	67.1 ± 10.8
BMI (kg/m^2^)	25.0 ± 3.0
Comorbid conditions	
• HTN	47 (70.1)
• DM	41 (61.2)
• CHF	9 (13.4)
• CKD	9 (13.4)
• CVD	12 (17.9)
Record duration (h)	5.2 (4.4, 6.1)
Procedure type	
• Conventional	53 (79.1)
• Minimally invasive	14 (20.9)
LVEF <40%	10 (14.9)
Vasoactive/inotropic agent use	
• Vasopressin	16 (23.9)
• Norepinephrine	65 (97.0)
• Dobutamine	24 (35.8)
• Milrinone	46 (68.7)
• Nitroglycerin	21 (31.3)
Intraoperative measurements	
• CO (L/min)	3.9 (3.4, 4.4)
• CI (L/min/m^2^)	2.3 (2.1, 2.5)

Values are mean ± SD, median (1Q, 2Q), or number (%). Abbreviations: BMI, body mass index; HTN, hypertension; DM, diabetes mellitus; CHF, congestive heart failure; CKD, chronic kidney disease; CVD, cerebrovascular disease; CO, cardiac output; CI, cardiac index.

**Table 2:** Selected features and their descriptions.

Feature	Description
dPoint	The amplitude of the dicrotic notch of the PPG waveform.
plethPerf	The average perfusion index, indicating blood flow strength at the sensor site in the PPG waveform.
diff2Quant25	The 25th percentile of the second derivative of the PPG waveform, reflecting signal curvature.
plethHr	The average heart rate calculated from the PPG waveform.
Ta1	The time interval from the foot of the PPG waveform to the time at which the first maximum peak from the first derivative of the PPG occurred.
Featb2	The minimum peak from the second derivative of the PPG waveform.
diff2SpecEntropy	The spectral entropy of the second derivative of the PPG waveform.
diff2IQR	The interquartile range of the second derivative of the PPG waveform.
diff1SpecEntropy	The spectral entropy of the first derivative of the PPG waveform.
c1	The first maxima point on the second derivative of the PPG waveform after the systolic peak.
tpt	The total time span of the PPG waveform.
plethSkewness	The skewness of the PPG waveform’s amplitude distribution.
plethKurtosis	The kurtosis of the PPG waveform’s amplitude distribution.
plethSat	The average oxygen saturation level derived from the PPG signal.
systPeak	The amplitude of the systolic peak on the PPG waveform.
plethPeakRms	The peak to RMS value of the PPG waveform.
plethSpecEntropy	The spectral entropy of the PPG waveform.
fftMean	The mean value of the Fast Fourier Transform (FFT) spectrum of the PPG waveform.
areaTotal	The total area under the PPG waveform curve.
area2	The area under the PPG waveform curve between two specific points: systolic point and dicrotic notch point.

**Table 3:** Detailed classification results by category in test set.

Category	Total Cases	Correct Predictions	Precision	Recall	F1-Score
Low CO	537	416	0.87	0.77	0.82
Non-low CO	943	880	0.88	0.93	0.91
Overall	1480	1296	0.87	0.87	0.87

**Table 4:** Comparison of recent studies on cardiac output estimation and classification.

Study	Methodology	Data Source	Performance Metrics	Findings
Xu et al. [44]	Improved U-Net model with PPG and ART signals	MIMIC dataset	Bias: −0.04 L/min, RMSNE: 10.0%	Demonstrated strong correlation with reference CO, using minimally invasive PPG and ART data.
Hong et al. [46]	Machine learning models for LCOS prediction	Clinical data from cardiac surgery patients	AUC (RF model): 0.909	Developed models highlighting 11 features to predict LCOS post-cardiac surgery.
Ke et al. [47]	ML algorithms with APW for CO estimation	MIMIC II waveform database	MSE (RF model): 1.421 L/min	Compared ML models for CO prediction, with RF showing strong accuracy and minimal bias.
Chiu et al. [45]	Classifiers for CO prediction from PPG	PhysioNet MIMIC-III dataset	Accuracy: 88.2%	Accurate classification of CO into low, healthy, and high ranges without calibration.
This Paper	Classification CatBoost algorithm with PPG signals	Off-pump coronary artery bypass surgery	Accuracy: 87.57%	Achieved high accuracy using only PPG signals, demonstrating efficiency in resource-constrained settings.

Abbreviations: PPG, photoplethysmography; ART, arterial pressure waveform; CO, cardiac output; LCOS, low cardiac output syndrome; ICU, intensive care unit; MSE, mean squared error; RMSNE, root-mean-squared normalized error; AUC, area under the curve; ML, machine learning; APW, arterial pulse waveform; RF, random forest.

## Data Availability

The data presented in this study are available on request from the corresponding author due to privacy concerns and ethical restrictions.
